# The Structure-Function Relationship between Macular Morphology and Visual Function Analyzed by Optical Coherence Tomography in Retinitis Pigmentosa

**DOI:** 10.1155/2013/821460

**Published:** 2013-12-03

**Authors:** Chang Ki Yoon, Hyeong Gon Yu

**Affiliations:** Department of Ophthalmology, Seoul National University College of Medicine, 103 Daehakro, Jongnogu, Seoul 110-799, Republic of Korea

## Abstract

*Purpose.* To evaluate the relationship between macular microstructures and visual function in retinitis pigmentosa (RP). *Method.* Fourier domain optical coherence tomography (FD-OCT) and Goldmann perimetry were used to examine 100 eyes of 100 RP patients. The preserved photoreceptor outer segment (PROS) length was measured at the horizontal and vertical high definition line scans. The PROS area was calculated from slab image and line scans simultaneously. The visual field area (VFA) was quantified. Each retinal thickness was measured: inner retina (IRT), outer retina (ORT), subfoveal choroidal thickness (SFCT), and central retinal thickness (CRT). *Results.* The PROS area values acquired differently were consistent. The VFA was related significantly to the CRT, ORT, PROS length (vertical and horizontal), and PROS area (line scan and slab image). Visual acuity was correlated with the CRT, ORT, IRT, PROS length (horizontal and vertical), and PROS area (line scan and slab image) significantly. Multiple linear regression analysis revealed that the PROS horizontal length and ORT were related to the VFA and visual acuity, respectively. *Conclusion.* Among the macular microstructures, the PROS horizontal length and the ORT were most correlated with VFA and visual acuity, respectively. However, SFCT is not related to visual function.

## 1. Introduction

Retinitis pigmentosa (RP) is a hereditary degenerative disorder characterized by progressive loss of photoreceptor and visual function. The visual field constricts as the disease progresses, eventually leading to severe visual loss in the advanced stage. Although visual field testing is used as a standard method to assess the progression of RP, several electrophysiological, psychophysical, and imaging techniques such as full-field electroretinogram (ERG), multifocal ERG, microperimetry, and autofluorescence imaging are being widely studied. The Goldmann visual field (GVF) is preferred for documenting the progression of RP because it is able to probe peripheral visual field constriction, which cannot be detected by automated perimeters, in the early phase. However, the large test-retest or intervisit variability of GVF, which is increased up to 50% in pathologic states like RP, makes it difficult to use this technique to evaluate disease progression or to assess the treatment outcome [1].

On the other hand, structural assessment using optical coherence tomography (OCT) offers high reproducibility. Even in RP, macula thickness and retinal nerve fiber layer (RNFL) analyses show high reproducibility [2]. The recent introduction of Fourier domain OCT (FD-OCT) has improved image resolution and has enabled the analysis of various macular morphologies quantitatively. RNFL thickness measurement is widely performed by using FD-OCT, and analysis of ganglion cell complex thickness has been adopted recently in glaucoma detection and progression evaluation. Previous RP studies revealed that the photoreceptor inner segment/outer segment (IS/OS) length and foveal thickness, especially the outer retinal thickness, are related to retinal functions such as the visual field, visual acuity, and ERG [3–5].

Although many studies have revealed the structure-function relationship of RP by using OCT, a systematic comparison to determine which structural parameter is more strongly related to visual function has not been performed. Therefore, we attempted to determine the retina microstructure that reflects visual function mostly in the point of vision and the visual field. We measured the preserved outer retinal layer in 3 dimensions, including horizontal/vertical length, thickness, and area. Further, choroidal thickness was evaluated to reveal its correlation with visual function.

## 2. Materials and Methods

### 2.1. Patients

Patient data were collected from the outpatient clinic at Seoul National University Hospital from 2008 to 2012. This study was approved by the Institutional Review Board at Seoul National University Hospital.

A total of 100 patients with clinically and electrophysiologically confirmed RP were included. The diagnosis of RP was based on a history of night blindness, impairment in peripheral visual fields, reduced amplitudes of rod and cone electroretinograms (ERG), and the presence of characteristic fundus pigmentary changes. All patients underwent a comprehensive interview and ocular examination including best-corrected visual acuity (BCVA), slit lamp biomicroscopy, dilated fundus examination, ERG, visual field testing, and OCT. The BCVA was evaluated by using a Snellen projection chart.

### 2.2. OCT Image Analysis

FD-OCT was obtained in all patients by using Cirrus HD-OCT (Carl Zeiss Meditec Inc., Dublin, CA, USA). The acquisition protocol consisted of an HD 5-line raster scan composed of 1024 A-scans at each 6 mm line and a macular cube with a 200 × 200 scan pattern in which a 6 mm × 6 mm region of the retina was scanned [6, 7].

OCT data with signal strength lower than 6 were excluded. Further, we excluded fully advanced cases in which the ELM and the PROS were not discriminated as well as patients who had macular edema and vitreomacular traction. All OCT images were acquired through a dilated pupil.

**Figure 1:**
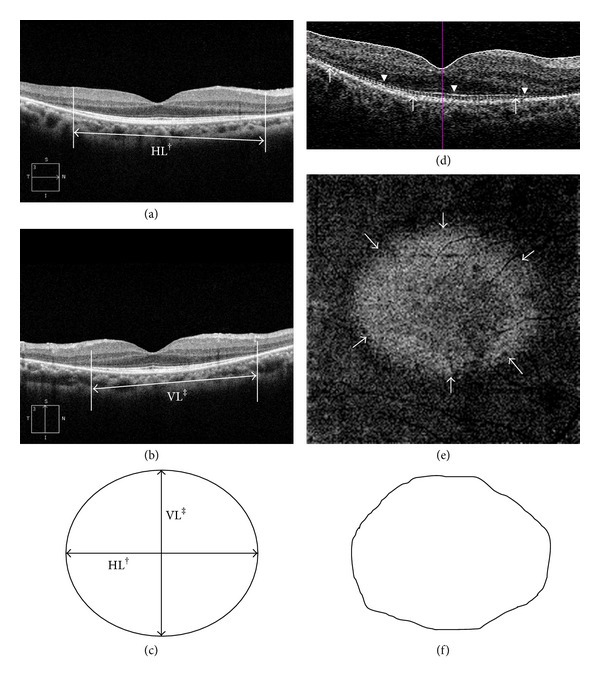
Representative image showing 2 methods for calculating the PROS area in the same patient. The PROS area measurement method using the horizontal and vertical PROS lengths of the high-definition (HD) scan is illustrated in (a)–(c), and the method using an en-face image of advanced visualization is demonstrated in (d)-(e). Horizontal HD scan (a) and vertical HD scan (b) of the same patient. The arrow indicates the PROS length (†: horizontal PROS length and ‡: vertical PROS length). The presumptive PROS area derived from the horizontal and vertical PROS lengths. Area of ellipsoid = *π*  × (horizontal length/2) × (vertical length/2) (c). Horizontal scan showing the slab layer (d). The dotted line marked by the arrowheads is the slab layer. The arrowheads indicate the photoreceptor IS/OS junction. An en-face image of the slab layer (e). The brighter region (surrounded by the arrows) is the preserved PROS area, and the darker region is the area where the PROS was not detected by OCT. Manual demarcation of the PROS area was carried out by using Photoshop CS 3.0 (f).

The preserved PROS area was calculated in 2 different ways. First, we determined the PROS area from the en-face image by using the advanced visualization option in Cirrus OCT 6.0 software, as previously described by Comander et al. for solar maculopathy [8]. The en-face PROS image could be visualized by using the slab image fit to the RPE layer. The width and location of the slab were finely tuned by viewing the cross-sectional image. Subsequently, the margin of the PROS was demarcated manually and the area was calculated by using Adobe Photoshop CS 3.0 (Adobe Systems Inc., San Jose, CA, USA) and Image J software (National Institutes of Health). Patients with a PROS area exceeding the 6 mm cube scan were excluded because the actual area of the preserved PROS could not be calculated. However, because an en-face PROS image was available for 64% of the patients, we obtained the PROS area alternatively by calculating the elliptical area derived from the horizontal and vertical PROS lengths measured by using the horizontal and vertical high-definition scan across the fovea. A representative image is shown in Figure 1. These cases were excluded if the preserved PROS was not within the 6 mm cube scan. We compared the areas measured by using these 2 different methods.

The CRT is the average thickness of the region from the ILM and RPE in a 1 mm circle, which is obtained by using macular thickness analysis. The inner retinal thickness (IRT) was defined as the distance between ILM and ELM, and the outer retinal thickness (ORT) was defined as the distance between ELM and inner border of RPE. Finally, the SFCT was defined as the distance between outer border of the RPE and inner border of the scleral wall. The IRT, ORT, and SFCT were measured manually at the thinnest point of the fovea by using the internal caliper provided in Cirrus OCT software. The average value from the horizontal and vertical image was used for statistical analysis.

### 2.3. Visual Field Analysis

The GVF test was performed by using the test target III4e under standard conditions on the same calibrated perimeter. The GVF was quantified according to a previously described method [9]. The extent of the centrally preserved visual field was obtained by computing the area surrounded by isopter polygons on scanned images of the perimeter charts and by calculating the solid angle subtended. The central visual field was defined as the region encompassing the central fixation, and the extents for the visual field of the scotoma were subtracted. We excluded the peripheral visual field island because an isolated preserved IS/OS junction in the periphery is difficult to detect by OCT. The visual field area (VFA) is reported as a percentage of the mean normal visual field [10]. This GVF extent unit, which is called the normalized solid-angle unit (nsu), was log-converted for comparison with other parameters, as described in the previous literature [9].

### 2.4. Statistical Analysis

Data from the right eye of each patient was used. All values were transformed to the log scale for statistical analysis because RP is known to progress exponentially [11]. The Pearson correlation was used to examine the strength of the association between visual function (LogMAR visual acuity and VFA) and each of the OCT morphology parameters (PROS area, PROS length, CFT, IRT, ORT, and SFCT). Multiple linear regression analysis was performed to determine the predictors of RP progression. Statistical analysis was performed by using SPSS 19.0 (SPSS Inc., Chicago, IL, USA).

## 3. Results

We included 100 eyes of 100 patients for analysis. The clinical characteristics are listed in Table 1. The average visual acuity was 20/30 (median, 20/28) and the average visual field was 22° (median, 10°).

**Figure 2:**
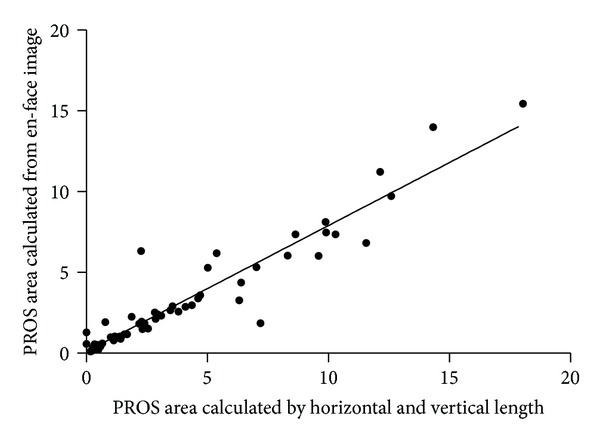
Scatter plot showing the PROS area measured by 2 different methods. The *X*-axis depicts the area calculated from the horizontal and vertical PROS lengths measured at each cross-sectional, high-definition image. The *Y*-axis depicts the area calculated from an en-face image in the advanced visualization tool (Pearson correlation: *ρ* = 0.953, *P* < 0.001).

A strong linear correlation was found between the 2 methods used for obtaining the PROS area (*r* = 0.953, *P* < 0.001) in 64 patients (Figure 2). We used the PROS area calculated by utilizing the horizontal and vertical lengths for comparison with the other parameters.

**Figure 3:**
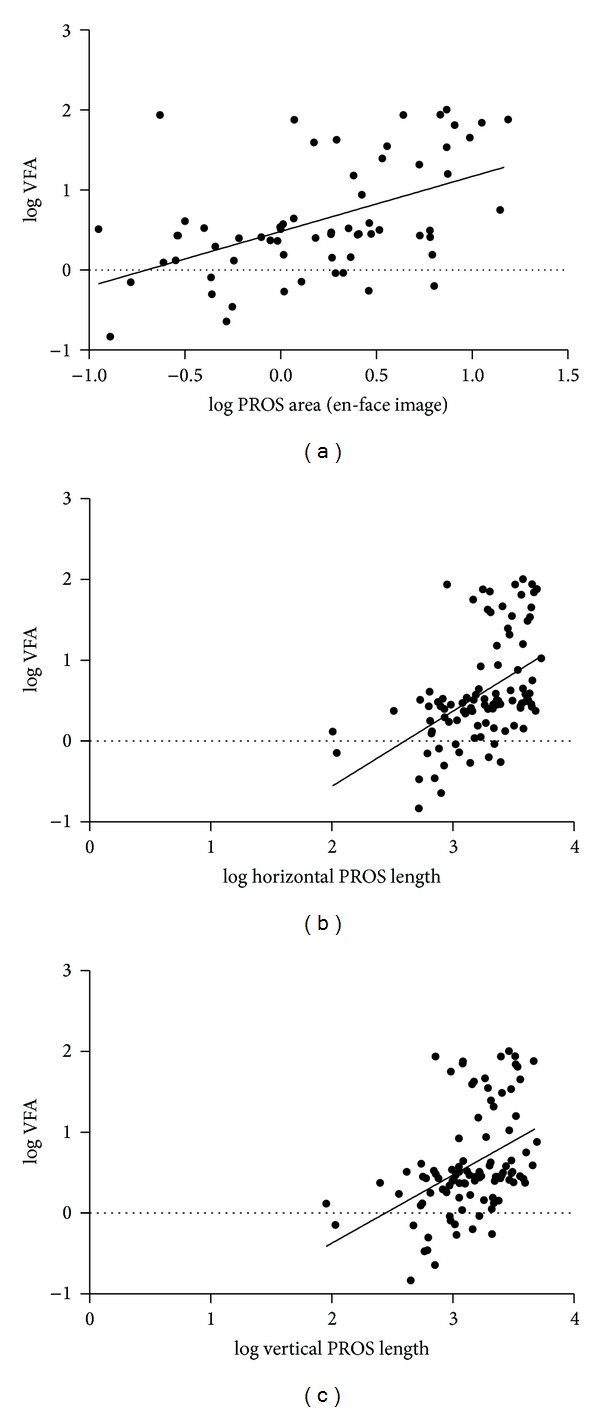
Scatter plot showing the relationships between the log PROS area (en-face image) and log visual field area (a), between the log horizontal PROS length and log visual field area (b), and between the log vertical PROS length and log visual field area. Solid line: the prediction of a linear model.

In univariable analysis using the Pearson correlation, the VFA was related to the horizontal PROS length (*ρ* = 0.483, *P* < 0.001), vertical PROS length (*ρ* = 0.426, *P* < 0.001), and PROS area (calculated from the horizontal and vertical lengths: *ρ* = 0.459, *P* < 0.001, and from the en-face image: *ρ* = 0.509, *P* < 0.001). The correlation between the log VFA and PROS length/area is presented in Figure 3. Regarding the thickness of the segmented retinal layer, the CRT (*ρ* = 0.276, *P* < 0.005) and ORT (*ρ* = 0.412, *P* < 0.001) were related to the VFA. The SFCT and IRT were not associated with the visual field. Generally, the correlation coefficient was higher for the PROS than for the retinal thickness. The PROS area calculated by using the en-face image showed the strongest relationship to the visual field.

**Figure 4:**
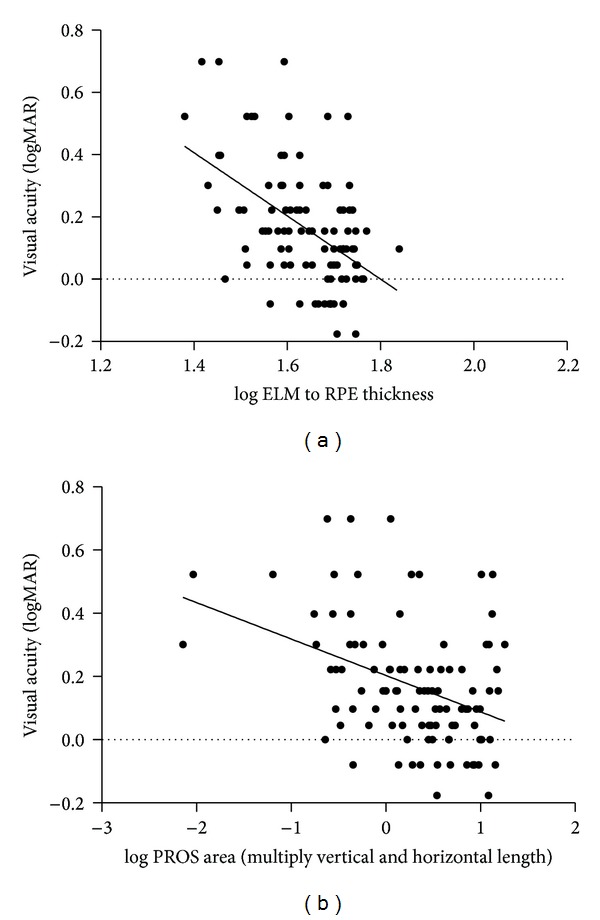
Scatter plot showing the relationships between the log outer retinal thickness (ELM to RPE) and logMAR visual acuity (a) and log PROS area (multiple vertical and horizontal lengths) (b). Solid line: the prediction of a linear model.

Visual acuity was correlated with the CRT (*ρ* = −0.339, *P* = 0.001), ORT (*ρ* = −0.519, *P* < 0.001), and IRT (*ρ* = −0.297, *P* = 0.003). The PROS horizontal length (*ρ* = −0.395, *P* < 0.001), PROS vertical length (*ρ* = −0.376, *P* < 0.001), and PROS area (calculated from the horizontal and vertical lengths: *ρ* = −0.389, *P* < 0.001, and from the en-face image: *ρ* = −0.370, *P* = 0.003) were also related to visual acuity. The ORT was most correlated with visual acuity (*ρ* = −0.519, *P* < 0.001). The correlations for visual acuity, ORT, and the PROS area are shown in Figure 4. The SFCT was not correlated with visual acuity. The correlations between visual function (visual field test and visual acuity) and the measured parameters (PROS values and retinal thickness parameters) are summarized in Table 2.

In multiple linear regression analysis, the horizontal PROS length was related to the VFA (*P* = 0.038). Further, the ORT was associated with visual acuity (*P* < 0.001).

## 4. Discussion

This study is the first to use the PROS area to estimate the progression of RP. To this end, 2 methods were used: an en-face image from an advanced visualization option and a presumptive ellipsoid derived from the horizontal and vertical PROS lengths. The former method is presumably more accurate and intuitive, but it could not be used in approximately 30% of the patients (36 patients). An en-face PROS slab image was usually not available in cases of high myopia. Because both methods showed consistent results, we used the PROS area determined from the horizontal and vertical lengths to identify the relationship to visual function.

The PROS area was significantly correlated with the VFA and visual acuity. These results are consistent with data suggesting that the photoreceptor IS/OS junction length is related to the focal ERG amplitude and Goldmann perimetry isopter [4, 5, 12]. The PROS area is expected to be a better parameter than the cross-sectional PROS length because it reflects the total preserved PROS amount. However, the horizontal PROS length showed a slightly better correlation with VFA than the PROS area calculated from the PROS length, and multiple linear regression analysis showed that PROS horizontal length was related to the VFA. Moreover, the horizontal PROS length is easier to measure than is the area. Considering the intervisit variability of the GVF test, the preserved PROS horizontal length can be a good progression index of RP. Further research is required to confirm the test reliability and long-term follow-up of the PROS length by using OCT.

Our data regarding the relationship between each retinal layer thickness and visual functions are consistent with previous studies [13–17]. In concordance with Lenassi et al. and Rangaswamy et al. [14, 15], although the total subfoveal thickness was related to visual acuity, it had a weaker correlation than the outer segment thickness. Further, multivariable linear regression analysis weighted the outer retinal thickness, and only the ORT was related to the VFA. Instead of evaluating the IS/OS junction to the RPE distance, we used the ELM-to-RPE distance as the ORT because this value was greater than the IS/OS-to-RPE height and was therefore expected to provide a smaller error than the PROS thickness. Moreover only the ELM is detectable even after the cone outer segment tip line and the IS/OS are disorganized in advanced RP [18, 19]. Thus, the ELM-RPE thickness value is more reliable for determination of outer retinal thickness in RP.

We analyzed the subfoveal choroidal thickness and found that it was not related to visual acuity or to the visual field. To our knowledge, this is the largest study to evaluate choroid thickness and to compare it with visual acuity and the visual field. The correlation between the SFCT and visual acuity was evaluated in 2 previous studies. Dhoot et al. reported that the SFCT was not related to visual acuity [20], whereas Ayton et al. reported that the SFCT showed a significant negative correlation with visual acuity [21]. We believe that our findings are more reliable than are those of these 2 studies because our study sample was much larger. A limitation of our choroid thickness data is that we did not compare this value with a control group. Many studies found that choroid thickness is reduced in RP patients compared to healthy controls [20–22]. Our study showed that visual acuity and the visual field are not directly related to the choroid thickness. A larger study is required to elucidate the choroid pathology in RP.

In this study, we compared the correlation between macular microstructural parameters and the visual function. Although the PROS area from the en-face visualization showed the strongest association with the visual field, this method was not available in 1/3 of our cases. The second most correlated value, horizontal PROS length, has the advantage of easy acquisition. We anticipate that measuring the PROS length will be useful for evaluating preserved visual function in RP patients who retain central vision.

## Figures and Tables

**Table 1:** Demographic data.

	Average (SD) (min~max)
Number of patients	100 (M : F = 58 : 42)
Age (year)	38.9 (12.8) (16~79)
Best-corrected visual acuity	20/30 (20/30) (20/100 to 30/20)
Visual field area (nsu)^†^	13.3 (24.0) (0.2~100.0)
PROS area^‡^ (mm^2^)	4.1 (4.3) (0.0~18.0)
PROS horizontal length (*µ*m)	2202.2 (1341.6) (102~5363)
PROS vertical length (*µ*m)	1767.2 (1105.3) (90~4942)
Central subfoveal thickness^*∮*^ (*µ*m)	248.2 (40.2) (135–383)
ILM-ELM thickness^*||*^ (*µ*m)	133.2 (34.9) (45.5~284.0)
ELM-RPE thickness^¶^ (*µ*m)	65.4 (15.4) (27.0~96.0)
Subfoveal choroid thickness (*µ*m)	265.5 (67.8) (89.5~422.5)

^†^Normalized solid angle unit.

‡Photoreceptor outer segment (PROS) area calculated from horizontal and vertical PROS length.

^*∮*^Central subfoveal retinal thickness.

^*||*^Internal limiting membrane to external limiting membrane (inner retinal thickness).

^¶^External limiting membrane to retinal pigment epithelium (outer retinal thickness).

**Table 2:** Univariable analysis.

Parameters	Versus log VFA^¶^				Versus BCVA (log MAR)^#^			
	*r*	*r* ^2^	*P*	*n*	*r*	*r* ^2^	*P*	*n*
PROS area^†^	0.459	0.211	<0.001*	100	−0.389	0.151	<0.001*	100
PROS area^‡^	0.509	0.259	<0.001*	64	−0.370	0.136	0.003*	64
PROS horizontal length	0.483	0.233	<0.001*	100	−0.395	0.156	<0.001*	100
PROS vertical length	0.426	0.181	<0.001*	100	−0.376	0.141	<0.001*	100
CRT	0.276	0.076	0.005*	100	−0.339	0.115	0.001*	100
Inner retinal thickness^*∮*^	0.153	0.023	0.134	97	−0.297	0.088	0.003*	97
Outer retinal thickness^*||*^	0.412	0.170	<0.001*	97	−0.519	0.269	<0.001*	97
Choroid thickness	0.017	0.000	0.866	96	−0.100	0.010	0.331	96

*P < 0.05.

^†^Photoreceptor outer segment area (the horizontal PROS length and the vertical PROS length were multiplied).

^‡^Photoreceptor outer segment area (calculated from an en-face slab image).

^*∮*^Internal limiting membrane to external limiting membrane (inner retinal thickness).

^*||*^External limiting membrane to retinal pigment epithelium (outer retinal thickness).

^¶^Log-converted visual field area (nsu).

^#^Best-corrected visual acuity by logMAR scale.
